# Distinct Expression Patterns of CD69 in Mucosal and Systemic Lymphoid Tissues in Primary SIV Infection of Rhesus Macaques

**DOI:** 10.1371/journal.pone.0027207

**Published:** 2011-11-09

**Authors:** Xiaolei Wang, Huanbin Xu, Xavier Alvarez, Bapi Pahar, Terri Moroney-Rasmussen, Andrew A. Lackner, Ronald S. Veazey

**Affiliations:** Division of Comparative Pathology, Tulane National Primate Research Center, Covington, Louisiana, United States of America; University of Pittsburgh, United States of America

## Abstract

Although the intestinal tract plays a major role in early human immunodeficiency virus (HIV) infection, the role of immune activation and viral replication in intestinal tissues is not completely understood. Further, increasing evidence suggests the early leukocyte activation antigen CD69 may be involved in the development or regulation of important T cell subsets, as well as a major regulatory molecule of immune responses. Using the simian immunodeficiency virus (SIV) rhesus macaque model, we compared expression of CD69 on T cells from the intestine, spleen, lymph nodes, and blood of normal and SIV-infected macaques throughout infection. In uninfected macaques, the majority of intestinal lamina propria CD4+ T cells had a memory (CD95+) phenotype and co-expressed CD69, and essentially all intestinal CCR5+ cells co-expressed CD69. In contrast, systemic lymphoid tissues had far fewer CD69+ T cells, and many had a naïve phenotype. Further, marked, selective depletion of intestinal CD4+CD69+ T cells occurred in early SIV infection, and this depletion persisted throughout infection. Markedly increased levels of CD8+CD69+ T cells were detected after SIV infection in virtually all tissues, including the intestine. Further, confocal microscopy demonstrated selective, productive infection of CD3+CD69+ T cells in the intestine in early infection. Combined, these results indicate CD69+CD4+ T cells are a major early target for viral infection, and their rapid loss by direct infection may have profound effects on intestinal immune regulation in HIV infected patients.

## Introduction

It is increasingly clear that the pathogenesis of HIV/SIV infection and AIDS is closely related to the activation state of the host immune system, and the immunologic and virologic events that occur during the earliest stages of infection may have a strong impact on disease progression [1], [2], [3], [4]. However, the relationship between the immune activation status of the host, viral replication, and infection or loss of specific immunoregulatory cells in tissues is not completely understood. Further, considerable debate exists as to what molecular markers truly define “activated” versus “resting” cells, particularly in mucosal tissues, and increasing evidence suggests CD69, previously considered to be an early activation marker, may also play a major role in immune regulation [5], [6], [7].

Several immunophenotypic markers have been used to evaluate the level of lymphocyte activation, including CD69, HLA-DR, CD25 (interleukin-2 (IL-2) receptor), CD38, Ki-67, and CD95. Of these, CD69 has been identified as the earliest activation marker on the surface of antigen- or allergen-specific activated lymphocytes, preceding the appearance of HLA-DR, CD25 and CD71 (transferrin receptor) [8]. Further, CD69 has been shown to be selectively expressed in chronic inflammatory infiltrates, and at the sites of active immune responses *in vivo* [9]. Although the specific role(s) of CD69 *in vivo* is not fully known, *in vitro* studies suggest it may act as a co-stimulatory molecule for T-cell activation and proliferation [10]. Moreover, CD69 is rapidly expressed upon T-cell activation in response to various stimuli, and is readily amenable to detection by immunofluorescence and flow cytometry, increasing its utility as a rapid response marker in assays of immune activation [11], [12]. Other activation markers are only upregulated late after activation (CD25, HLA-DR) and/or associated more with antigen experience (CD95) or cell proliferation (Ki-67) than activation *per se*.

Most of our evidence for CD69 as an activation marker comes from assays based on responses of peripheral blood lymphocytes. For example, CD69 is considered an early activation marker as it is rapidly upregulated on essentially all bone marrow derived lymphocytes following in vitro stimulation [5], [13]. However, CD69 is also transiently expressed on developing T lymphocytes in the thymus [14], [15], [16] and recent evidence suggests there may be multiple roles for CD69 expression including immune regulation, inflammation, trafficking, and possibly even in the development of anergy or tolerance [5], [6], [7]. Thus, while CD69 is clearly associated with early cell activation, its role may differ in various tissues or cellular microenvironments. Further, more recent evidence suggests CD69 may directly influence the development of Treg CD4+ T cells, enhance differentiation of Th17 cells [13], and regulate TGF-beta secretion [13], [17], and thus may play a major role in regulating immune responses to infections such as SIV/HIV.

Studies have consistently confirmed that memory CD4+ T cells are rapidly depleted in tissues of SIV and HIV-infected hosts, and this depletion is much more profound in the intestine than in peripheral lymphoid tissues [18], [19], [20], [21], [22]. Although it has been proposed that this rapid and dramatic loss of intestinal CD4+ T cells is due to the high proportion of “activated” memory CD4+ T cells expressing CCR5 which are more permissive of viral infection [23], the mechanisms by which these cells are eliminated remain controversial. Here we evaluated the expression of CD69 on T cell subsets from various tissues of normal rhesus macaques, and assessed dynamic changes in activated T cells throughout SIV infection.

## Results

### Distribution of CD69+ CD4+/CD8+ T cells in tissues of normal rhesus macaques

**Figure 1:**
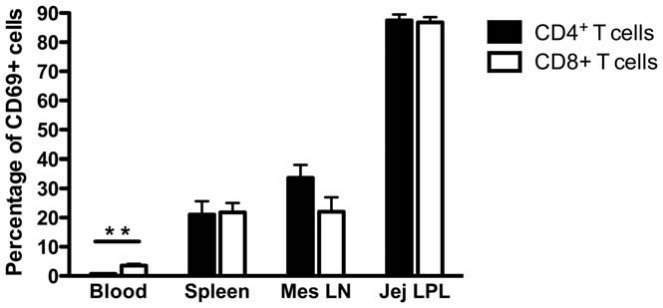
Expression of CD69 on CD4+ and CD8+ T cell in tissues of normal (non-infected) adult rhesus macaques. Note the intestine has far more CD69+ T cells than other peripheral lymphoid tissues, and T cells in blood lack CD69 expression. Bars represent mean percentages of CD69+ cells when gating through either CD3+CD4+ (black) or CD3+CD8+ (white) T cell subsets ± SEM. Significant differences between CD4 and CD8 are indicated by asterisks as; **P<0.01 using a Mann Whitney U test.

By flow cytometry, percentages of both CD4+ and CD8+ T cells co-expressing CD69 were markedly lower in blood compared to other tissues (**Fig. 1**) consistent with circulating T cells having a mostly “resting” phenotype. In contrast, most CD4+ T cells in the intestine co-expressed CD69 (mean 87%, range 80–95%), and a large percentage of CD4+ T cells in the mesenteric lymph node (33%) and spleen (21%) co-expressed CD69. Expression of CD69 on CD8+ T cells showed a similar distribution. Specifically, most CD8+ cells (86.9%) in the intestine co-expressed CD69, and to a lesser extent, spleen (mean 21.8%) and mesenteric lymph nodes (mean 22%). Only 3.6% of blood CD8+ T cells co-expressed CD69 (**Fig. 1**). Interestingly, CD69 expression on T cells was higher in mesenteric lymph nodes (GALT) than in axillary lymph nodes (systemic lymphoid tissue), but these differences were not significant (data not shown). Combined, these data indicated that intestinal sites and their draining tissues (mesenteric LN) have higher levels of CD69 expression than systemic tissues. In the blood, percentage of circulating CD8+ T cells co-expressing CD69 was higher than that of CD4+ T cells (P = 0.002) despite the overall low levels of CD69 expression in this tissue. However, there were no such significant differences between CD69 expression on CD4 and CD8 T cells in other tissues examined.

### Intestinal processing procedures do not artificially upregulate CD69 expression

**Figure 2:**
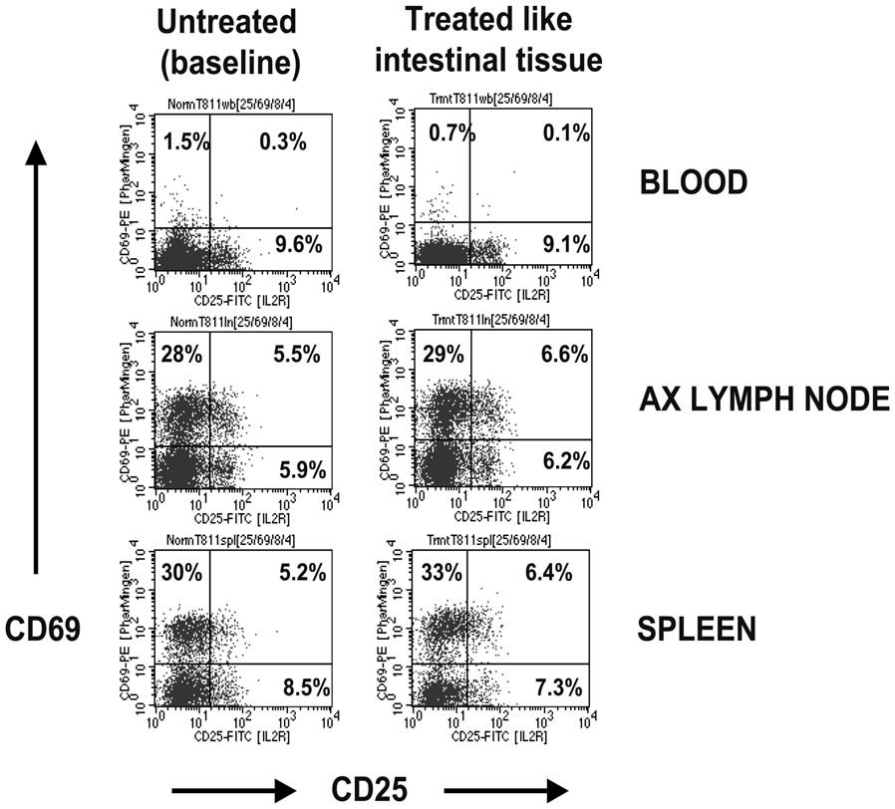
Flow cytometry plots showing CD69 and CD25 expression on lymphocytes from peripheral lymphoid tissues immediately ex vivo (left) and after treatment using the same EDTA/collagenase digestion procedures used to extract intestinal lymphocytes. Note that these procedures do not artificially up-regulate either activation markers on CD4+ T lymphocytes. Plots were generated by gating on CD3+ lymphocytes, and then CD4+ T cells. Data are representative of tissues from 5 animals performed in 3 separate experiments.

Procedures required to prepare single cell suspensions from the intestine require 2–3 hours to perform due to sequential incubation with a variety of chemicals and potential antigens including EDTA, collagenase, and fetal calf serum. Since CD69 is rapidly upregulated upon antigen or mitogen exposure in vitro, it was conceivable that these procedures could artificially upregulate CD69 ex vivo. To address this concern, we processed and treated PBMCs and lymphocytes from blood, lymph node, and spleen using identical procedures as used for intestinal cell isolation including serial incubations in media with EDTA followed by collagenase treatment exactly as above with rapid shaking at 37 degrees for the same time intervals. As shown in **Fig. 2**, there was essentially no increase in CD69 expression or changes in any other markers on T cells in the blood, lymph node or spleen after cells were subjected to identical treatments as intestinal cells. Therefore, the intestinal flow cytometry data we obtained from examining cell suspensions prepared from intestinal tissues are indeed representative of the cell phenotypes observed *in situ*.

### Intestinal CD4+T cells expressing CD69 have a “memory” phenotype

**Figure 3:**
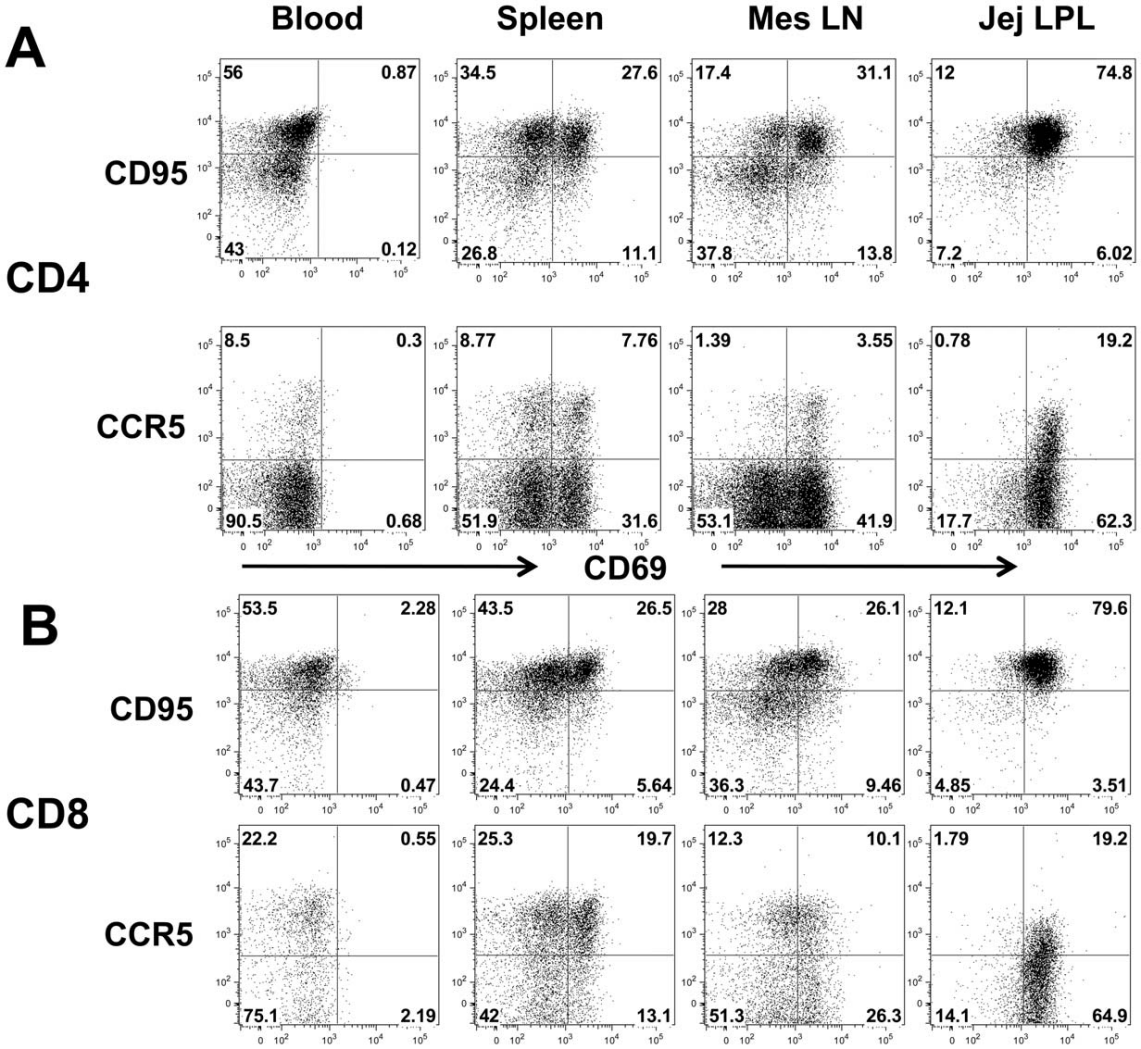
Phenotyping activated (CD69+) T cells using CD95 (memory marker) and CCR5 (HIV/SIV co-receptor) expression on CD4 or CD8+ T cell subsets from various tissues of normal rhesus macaques. Note the vast majority of the memory CD95+CD4+ (A) and CD95+CD8+ (B) T cells in the intestine co-express CD69+, while far fewer memory cells co-express CD69 in other tissues. Also note CCR5 expression is limited to CD69+ T cells in the jejunum, but expressed on both CD69+ and CD69 negative cells in other tissues. Plots were generated by gating through CD3+ lymphocytes and then CD4+ or CD8+ T cells.

To further characterize and phenotype CD69+ T cells subsets, we examined the co-expression of CD95 (memory marker) and CCR5 (major co-receptor for HIV/SIV) on CD69+ CD4+ and CD8+ T cells. Significant differences in levels of co-expression of CD95 and CCR5 were detected on CD4 and CD8+ T cells between tissues of normal rhesus macaques. Nearly all CD69+CD4+ T cells in the intestine lamina propria co-expressed CD95, yet many mesenteric LN, and even more spleen CD69+CD4+ T cells lacked CD95 co-expression (**Fig. 3A**). In addition, CCR5 expression was differentially expressed on CD69+ T cells in tissues as essentially all intestinal CD4+ T cells that co-expressed CCR5 also expressed CD69, yet CCR5 was expressed on both CD69+ and CD69neg T cells in mesenteric LN and spleen. In contrast, neither CD95+ nor CCR5+ T cells in blood co-expressed CD69. Generally, CD8+ T cells showed similar patterns for co-expression of CCR5 and CD95 (**Fig. 3B**). Combined, these data show that CD69 expression is differentially expressed on naïve and memory T cell subsets in tissues, and that the vast majority of CD4+CD69+ T cells in the intestinal lamina propria have a memory phenotype (CD95+). Further, these data confirm that the vast majority of these activated CD4+ T cells in the intestine are memory and many co-express CCR5, and are thus optimal target cells for HIV infection and replication.

### Examining CD69 expression in tissues (*in situ*)

**Figure 4:**
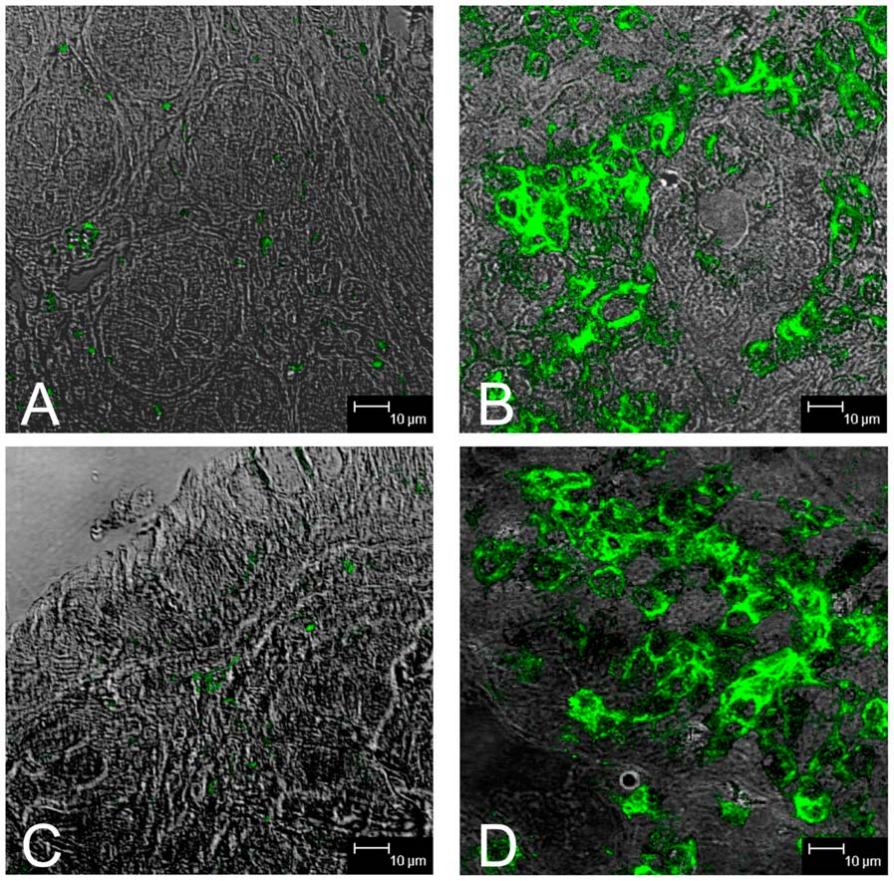
Comparison of immunohistochemistry results on formalin fixed (left) and tissues. Figures A and C show CD69 expression in formalin-fixed, paraffin-embedded intestinal tissues and B and C are snap frozen intestinal tissue sections from adjacent regions of tissue from the same animal. Note that markedly higher numbers of CD69+ cells are detected when using frozen tissues.

To visualize CD69 positive cells in tissues, and determine if such activated T cells are the initial targets for SIV infection, we attempted immunohistochemistry and immunofluoresence for CD69 on formalin-fixed, paraffin embedded tissue sections following routine protocols. However, in contrast to the flow cytometry data, very few CD69+ cells were detected in formalin-fixed tissues (best staining is represented in **Fig. 4A and C**). Since formalin can cross-link proteins/antigens of interest, we attempted several different antigen retrieval methods that we have used successfully, including high-pressure steam, pressure cooking, proteinase K treatment, and high pH unmasking buffer treatment, none of which improved the detection of CD69 on formalin fixed tissues (data not shown). Thus, we hypothesized that formalin fixation irretrievably cross-linked CD69 expression in tissues, and thus resorted to snap frozen tissues for immunofluoresence and confocal microscopy analysis of CD69 expression *in situ*.

**Figure 5:**
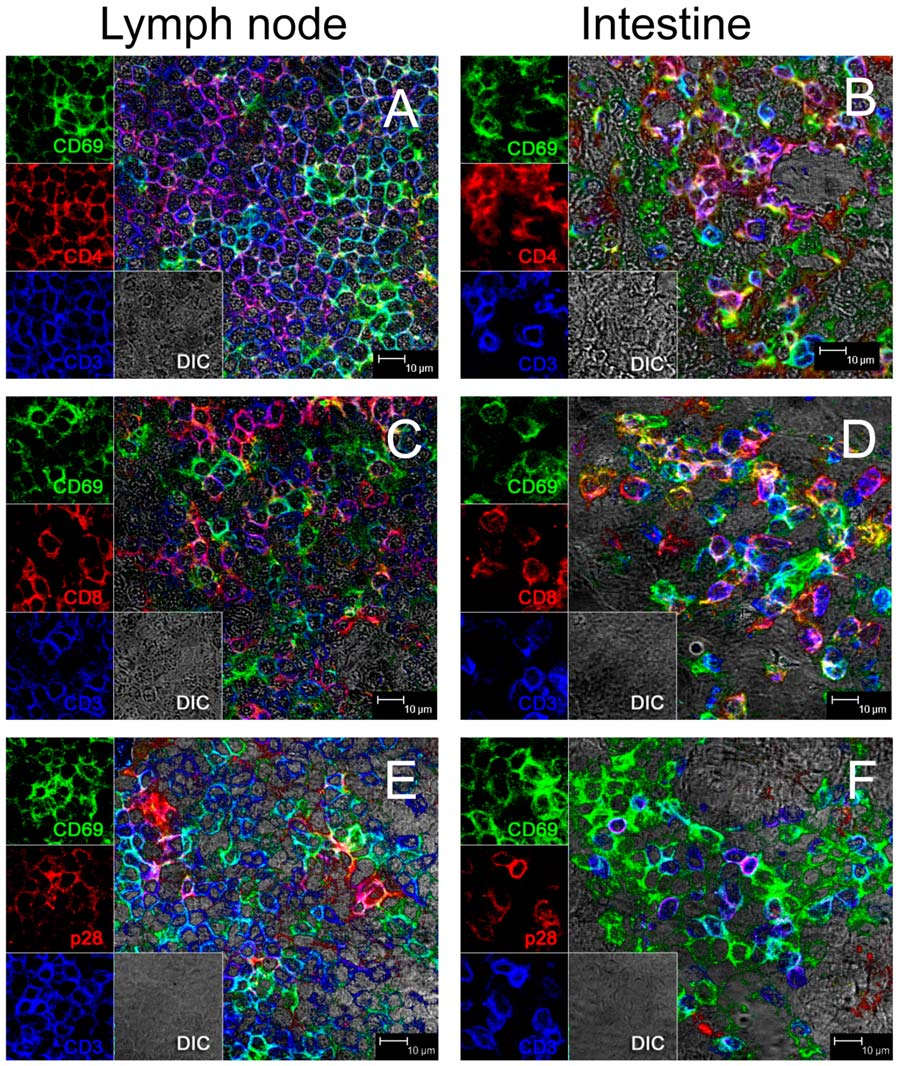
Phenotyping activated T cells in lymph node (left) and intestine (right) by multilabel immunofluoresence and confocal microscopy. Note that essentially all intestinal CD4+ (B) and CD8+ (D) T cells co-express CD69 (yellow/white), as most easily observed in images from individual channels on the left of each figure. Note there are no single positive “red” CD4+CD69neg cells in the intestine (B, D) but some are present in the lymph nodes (A, C) of . uninfected macaques. Panels E and F show productively SIV-infected cells as indicated by SIV p28 staining (red) and demonstrate most SIV-infected cells in tissues co-express CD3 and CD69 (purple) in both lymph node (E) and intestine (F) in early SIV infection. A single large “pure red” SIV+ cell not expressing CD69 or CD3 is visible in the upper left quadrant of E which may be a dendritic cell or macrophage. This macaque was examined 10 days after SIV infection.

Comparing the expression of CD69 on frozen sections obtained from adjacent blocks of snap-frozen tissue from the same animal, we found markedly higher CD69 expression on frozen sections (**Fig. 4B and D**) than on formalin fixed tissues (**Fig. 4A and C**). Further, CD69+ cells co-expressed either CD4 or CD8, and CD3 (**Fig. 5A, B, C and D**), consistent with the flow cytometry data on cell suspensions from the same animals. Combined, these findings demonstrated that detection of CD69 on formalin fixed, paraffin embedded tissues is problematic and that frozen sections must be used to evaluate CD69 expression *in situ*.

Using three color immunofluorescence and confocal microscopy, we simultaneously examined P28 antigen (SIV gag), CD69 and CD3 expression on frozen tissues from 3 early SIV infected macaques (8 d.p.i.), and found that most SIV-infected T cells (P28+CD3+) in both mesenteric lymph node (mean 62%) and jejunum (mean 80%) also co-expressed CD69 (**Fig. 5E and F**), verifying that CD69+ T cells were indeed major initial targets for SIV infection *in vivo*. Further, at least 70% of the SIV+ cells co-expressed CD3 but it was not determined whether the few CD3 negative SIV+ cells represented macrophages, dendritic cells, or even T cells that had down regulated CD3.

### Dynamics of CD69+ CD4+ and CD69+CD8+ T cells *in vivo* during acute SIV infection

**Figure 6:**
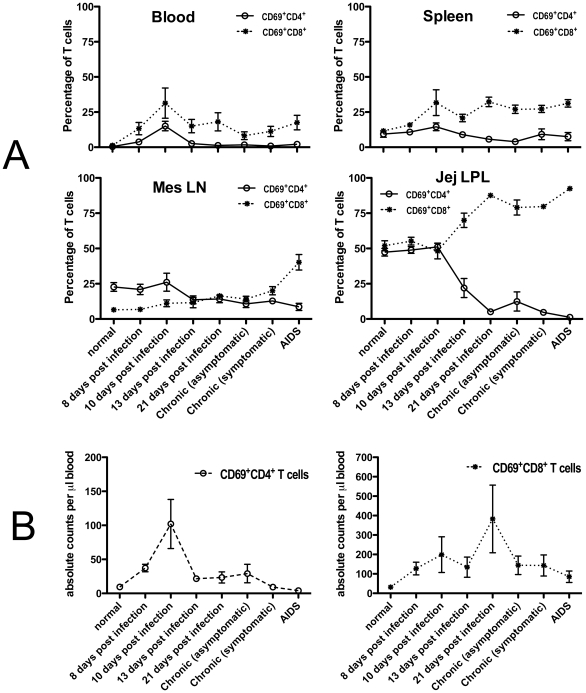
Changes in CD69 expressing CD4+ (solid lines) and CD8+ (dotted lines) T cells in various tissues after SIV infection expressed as (A) percentages of total T cells gated through CD3+ lymphocytes in tissues and (B) absolute counts of CD69+CD4+ and CD69+CD8+ T cells in blood. Note that levels of CD69+CD8+ T cells are increased in all tissues examined. In blood, CD4+CD69+ T cells are transiently increased at day 10, but rapidly decline to undetectable levels thereafter. In contrast, CD4+CD69+ T cells are rapidly, markedly, and persistently depleted in the intestine and to a lesser extent in the mes LN (bottom). Moreover, sustained increases in absolute counts of CD69+CD8+ T cells were found in the blood (6A), suggesting the increase in the percentage CD69+ CD8+ T cells showing in **Fig. 6A** is not due to the proportional loss of CD69+CD4+ cells in the CD3 gated populations. Combined, these data indicate CD4+CD69+ T cells are rapidly destroyed, whereas CD8+CD69+ T cells rapidly increase in SIV infection. This rapid, profound, and selective loss of intestinal CD69+CD4+ T cells is likely the result of direct viral infection as evidenced in Fig. 5. Data in A represent mean percentages of T cells gated first through CD3+ T lymphocytes (mean ±SEM).

To evaluate the effects of SIV infection on activated T cells *in vivo*, we also compared levels of CD69+ expression on T lymphocytes from the blood, spleen, mesenteric LN and intestine of animals at various stages of SIV infection. We found SIV infection had profound effects on both activated CD4 and CD8 T cells. As shown in **Fig. 6**, SIV infection resulted in significantly increased levels of CD69+CD8+ T cells in all tissues examined, including blood (P = 0.0072), spleen (P<0.0001), mesenteric lymph node (P<0.0001) and jejunum (P<0.0001). In blood, there was a transient yet significant (P<0.0001) increase in percentages (**Fig. 6A**) and absolute numbers (**Fig. 6B**) of CD69+CD4+ T cells at 10 days of infection, but this subsequently declined. Marked declines in CD4+CD69+ T cells were observed in jejunum (P<0.0001) and mesenteric lymph node (P = 0.002) by 13 days of infection and declined thereafter throughout infection. No significant differences in CD69+CD4+ T cells were detected in spleen (P = 0.29) between uninfected and SIV-infected macaques. Combined, these results indicate that there are markedly different responses between CD4+ and CD8+ T cells expressing CD69 in tissues; CD8+CD69+ cells generally increase, whereas CD4+CD69+ cells are rapidly and persistently depleted in tissues, particularly in GALT.

Although circulating T cells lack CD69 expression in normal macaques, both CD4+ and CD8+ T cells expressing CD69 increased in blood 10 days after SIV-infection. These CD69+ T cells mostly co-expressed CD95 (data not shown) indicating these were cells that had previously encountered antigens and were currently activated. These cells likely support direct SIV infection and may play a role in trafficking and dissemination of infected cells through the blood to tissues in very early infection.

### Correlation between viremia and T-cell activation SIVmac251 infected macaques

**Figure 7:**
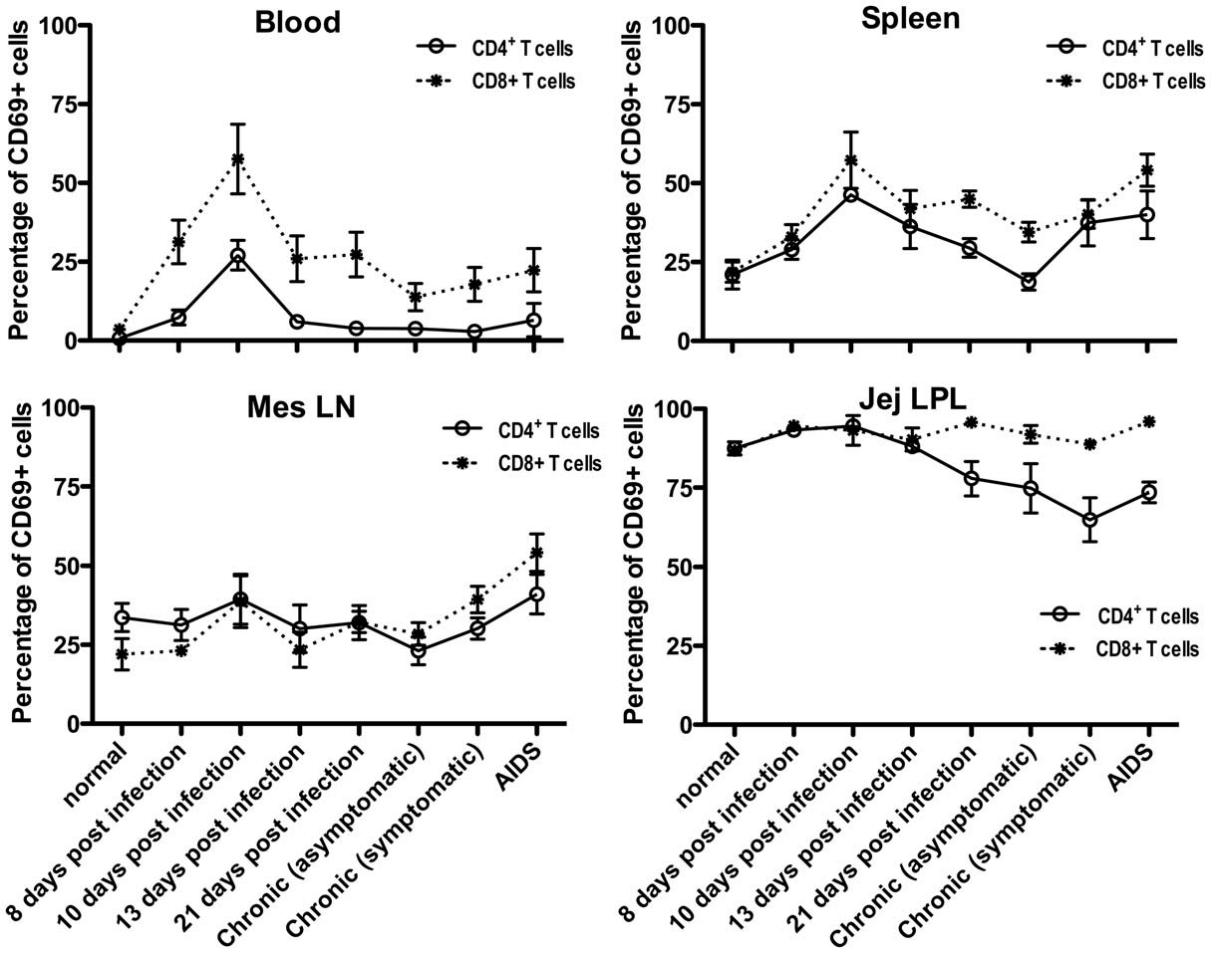
Changes in CD69 expression on individual CD4+ (solid lines) and CD8+ (dotted lines) T cell subsets from various tissues in SIV uninfected and infected macaques. Note that percentages of CD4+ T cells co-expressing CD69 spiked at day 10 post infection in all tissues and subsequently declined, until the onset of symptoms, when levels again started to rise (compare these to viral load data in Fig. 7). In contrast, CD4+CD69+ T cells in the jejunum declined after peak viremia. Data represent total mean percentages of CD69+ cells when gating through either CD3+CD4+ (black) or CD3+CD8+ (white) T lymphocytes (mean ±SEM).

As previously reported [5], [20], [24] we found that peak viral loads of 10^7^ to 10^8^ SIV RNA copies/ml were detected in plasma around 10 days of infection, then declined to a viral “set point”, until the onset of AIDS (data not shown). Further, plasma viral loads throughout SIV infection paralleled the levels of CD69+CD4+ T cells in examined tissues, especially in the intestine (Spearman r = 0.4, P = 0.01), and to a lesser extent, in blood (Spearman r = 0.3, P = 0.046) and spleen (Spearman r = 0.3, P = 0.041), suggesting levels of activated CD4 T cells closely correlate with plasma viremia. Moreover, gating through the remaining T cells revealed that the most profound changes in CD69 expression on T cell subsets were found in blood. As shown in **Fig. 7**, increased levels of CD69+ T cells were detected at 8 d.p.i. which peaked at 10 d.p.i., then declined after 13 d.p.i., but were still sustained at high levels through primary infection. This suggests persistent immune activation in SIV infection is associated with progression to AIDS. Similar to activated (CD69+) CD4+ T cells, a positive correlation was found between plasma VL and CD8+CD69+ cells in blood (Spearman r = 0.35, P = 0.03). However, changes in CD69 expression on T cells in other tissues differed, especially in intestine. Specifically, despite large numbers of CD69+CD8+ T cells persisting in the intestine throughout SIV infection, percentages of remaining intestinal CD4 T cells expressing CD69 rapidly and continuously declined after 13 days of SIV infection (**Fig. 7**) again reflecting differential effects of SIV infection on CD4+ and CD8+ T cells. Further, a negative correlation was detected between activated CD8+ T cells and plasma VLs in both the jejunum (Spearman r = −0.4, P = 0.01) and mesenteric lymph nodes (Spearman r = −0.4, P = 0.02) suggesting mucosal or GALT CD8+ T cell activation correlates with control of plasma viremia.

## Discussion

Both HIV and SIV cause rapid, persistent depletion of memory CD4+ T-cells soon after infection, suggesting early immunologic effects of infection may play a critical role in determining the subsequent disease. These and prior results have suggested rapid and selective depletion of intestinal CD4+ T cells was due to a combination of their state of activation and expression of high levels of CCR5 [20], [25], [26]. We originally proposed that cell activation, particularly in tissues plays a major role in viral replication, amplification, and systemic spread of infection through such activated cells [25], [27]. More recently, mucosal (gut) damage has also been shown to promote systemic immune activation fueling HIV-1 persistence and replication in systemic tissues as well [4], [28]. Thus, examining levels of immune activation in situ (tissues) is of critical importance for understanding these early events. However, markers of immune activation, and assessing levels of immune activation in vivo is controversial and sometimes problematic due to both cellular and viral dynamics and the technical limitations of tracking individual cells in vivo [29]. Moreover, emerging evidence indicating CD69 is a major immunoregulatory molecule involved in Treg and Th-17 cell development and regulation [13] merited further analysis of the dynamics of CD69 expression in early SIV infection. Examining the distribution and dynamics of CD69 expression in tissues, particularly in early SIV/HIV infection may provide important clues to the immune dysregulation that is associated with progression to AIDS.

Of known activation markers, CD69 has been shown to be the earliest marker for T cell activation. It is expressed by T cells within 30 min of T-cell receptor ligation, and is readily amenable to detection by immunofluorescence and flow cytometry [11], [12], [30]. Flow cytometry data revealed levels of CD69 expression markedly differed on T cells isolated from different tissue sites in uninfected macaques (**Figs. 1** **and** **3**). Further, both flow cytometry and immunohistochemistry (on frozen tissues) were concordant, demonstrating that intestinal lamina propria T cells are overwhelmingly CD69+. The co-expression of CD95 on all, and CCR5 on most CD69+ T cells in the gut suggests that these are activated memory T cells, rather than resting or naïve T cells. These data are consistent with the hypothesis that intestinal lamina propria T cells have increased states of activation, which may be the result of their continuous exposure to foreign antigens from the intestinal lumen. In other tissues however, many CD69+ cells did not co-express CD95 or CCR5, suggesting that differential regulation of CD69 expression may exist in different tissue sites. Circulating CD4+ T cells in the blood of normal macaques had minimal CD69 expression, yet substantial percentages of activated CD69+ T cells were detected in other lymphoid tissues (spleen and mesenteric lymph node). However, the latter tissues displayed very different patterns of CD95 and CCR5 expression on CD69+ T cell subsets. Conceivably, CD69 is only transiently expressed in peripheral tissues in response to early activation, and may be lost on cells once they migrate into the blood for recirculation and trafficking. Alternatively, CD69+ cells may reflect acutely activated cells that have not had time to convert to a memory phenotype. Regardless these data demonstrate that virtually all jejunum lamina propria CD4+ T cells are antigen-experienced memory cells (CD95+) and co-express CD69, suggesting they are still in an activated state. Conceivably however, CD69 in intestinal tissues may be associated with local immune regulation rather than early “activation”, since very few CD69 negative cells could be detected in this tissue.

Levels of CD69 expression on total T cells increased after SIV infection in most tissues examined, consistent with SIV infection inducing immune activation. However, we found markedly decreased levels of CD4+CD69+ T cells in the intestine and mesenteric LN of infected macaques, whereas markedly increased levels of CD8+CD69+ cells were detected in the same tissues. This differential response is strongly suggestive of a direct effect of SIV infection of CD4+ T cells rather than non-specific immune activation or bystander apoptosis, since activated CD8+ T cells are actually increased throughout infection (**Figs. 6** **,** **7**). Furthermore, phenotyping SIV infected cells in situ demonstrated marked and selective infection of CD69+ T cells in tissues, which is also consistent with direct infection of CD4+ T cells (**Figs. 5E and F**). Combined, these findings provide additional evidence that intestinal CD4+ T cells are highly susceptible targets for viral infection and lysis in early infection, and that activated CD4+ T cells are selectively depleted in primary infection. The dynamics of CD4+ T cell destruction and turnover in vivo, particularly in tissues, make it very difficult to definitively prove the mechanisms of CD4+ T cell destruction when examining “snapshots” of data collected at single timepoints [29]. However, the fact that CD3+CD69+ cells are infected at greater rates than CD3+CD69neg T cells throughout infection (**Fig. 5**), combined with the fact that CD8+CD69+ cells increase and CD4+CD69+ T cells decrease in most tissues (**Fig. 6**) strongly suggest that CD69+CD4+ T cells are lost through direct infection and lysis, rather than “bystander” apoptosis or chronic immune activation of uninfected cells. If activation-induced bystander apoptosis were involved, it would seem that CD8+ T cells would also be lost because they demonstrate even higher levels of activation (CD69+) than the residual CD4+ T cells, yet both proportions and numbers of activated CD8+CD69+ T cells show a sustained increase after SIV infection in all tissues, particularly in intestinal tissues (**Fig. 6**). Thus, we believe these data provide solid evidence that infected, activated CD4+ T cells are being destroyed, yet the actual mechanism of this loss (apoptosis versus lysis) will remain debated. Definitively determining the mechanism of CD4+ T cell depletion remains a central unresolved issue in AIDS research [**31**].

There is abundant circumstantial and *in vitro* evidence, yet a limited amount of direct experimental evidence, that immune activation drives HIV replication *in vivo*. Further, it is still unclear as to whether immune activation is a cause or consequence (or both) of viral replication in infected hosts, particularly since most of the activation is nonspecific for HIV/SIV antigens. Unfortunately, use of CD69 as an activation marker alone may not fully characterize the status of immune activation. In the intestine, CD69 appears to be constitutively expressed on CD4+ and CD8+ T cells, since few to no CD69 negative cells were detected in the lamina propria of the jejunum. This was further supported by the co-expression of CD95 on virtually all CD69+ cells in the intestine. Interestingly however, cells in other tissues did not reflect such concordant expression of CD95 and CD69 expression, suggesting different functions or regulation of CD69 expression in different tissue sites.

Although the expression of CD69 has been well documented in systemic cells (PBMC) the true function of CD69 and/or these “activated” T cells in the intestine remains uncertain, as increasing evidence shows multiple roles for CD69 as a potential immune regulator, possibly by, suppressing or dampening immune responses [17], [32] or even regulating the differentiation or production of key T cell subsets such as Th-17 and CD4+ Treg cells [13]. It is tempting to speculate that CD69+ cells in the intestine may be playing a role in the “oral tolerance” of the gut, a feature thought to be important in suppressing immune responses to “food” antigens. Further, “activated” T cells in the gut may simply have higher rates of turnover than those in circulation, and our data may reflect large percentages of newly arrived T cells that will soon be replaced by other activated T cells. Although we could not determine if these cells are being rapidly replaced or had suppressive functions, our combined data suggest that these are indeed antigen activated memory cells and are major targets for early SIV infection, and likely, subject to direct viral-mediated lysis.

Further, the observation that peak levels of CD69+ expression occur on both CD4+ and CD8+ T cells in the blood at 10 days post infection suggest primary SIV infection induces rapid systemic immune activation in response to infection, and thus provides even more activated CD4+ T cells, which HIV may use for additional infection, dissemination, and spread to other tissue sites in primary infection. Clearly, more studies are needed to validate these findings in HIV infected patients, but these results suggest that CD69 expression may play a major role in the early pathogenesis of HIV infection, and particularly in the rapid depletion of memory CD4+ T cells in tissues. Early infection and destruction of intestinal CD4+ T cells appears to be selective for memory CD4+ T cells co-expressing CD69, which are likely much more susceptible to infection through expression of appropriate co-receptors (CCR5) and their increased state of activation. Nonetheless, since emerging evidence indicates CD69 is a major regulator of immune responses and T cell development, selective loss of CD4+ T cells expressing CD69 may have profound consequences for local immune regulation in specific tissues.

## Methods

### Ethics Statement

The Institutional Animal Care and Use Committee (IACUC) of Tulane University approved all macaque procedures described (protocol permit number 3562). This study was carried out in strict accordance with the recommendations in the Guide for the Care and Use of Laboratory Animals of the National Institutes of Health (NIH) and with the recommendations of the Weatherall report; “The use of non-human primates in research”. All procedures were performed under anesthesia using ketamine, and all efforts were made to minimize stress, improve housing conditions, and to provide enrichment opportunities (e.g., objects to manipulate in cage, varied food supplements, foraging and task-oriented feeding methods, interaction with caregivers and research staff).

### Animals and virus

Rhesus macaques (*Macaca mulatta*) were obtained from and housed at the Tulane National Primate Research Center. Eight uninfected rhesus macaques were euthanized for tissue collection as controls, and another 37 were infected with SIVmac251 or SIVmac239 and euthanized for tissue collection at various time points, including very early (acute) at 8 days (n = 6), 10 days (n = 3), 13 days (n = 5) and 21 days (n = 4) post infection, or in chronic infection (defined here as infected over 42 days) with either no overt signs of disease (chronic asymptomatic, n = 6), or with illness that could not be definitively attributed to AIDS (e.g., nonresponsive diarrhea or weight loss; n = 6), and 7 animals with overt signs of AIDS, all of which had AIDS defining lesions and/or opportunistic infections including *Pneumocystis carinii* pneumonia (n = 4), disseminated *Mycobacterium avium* infection (n = 2) or SIV encephalitis (n = 1).

All animals examined in acute infection (21 days or less) were intravenously infected with 100 TCID_50_ SIVmac251 to reduce variation that can occur with mucosal inoculations, but macaques in chronic infection were either intravenously or intravaginally inoculated and grouped together irrespective of route of inoculation.

### Cell isolation and flow cytometry

Tissues for flow cytometry and immunohistochemistry were collected from the jejunum, spleen, mesenteric and axillary lymph nodes within minutes of necropsy and transported to the lab on ice for immediate processing. Lymphocytes from the intestine were isolated and stained for flow cytometry as previously described [19]. Briefly, intestinal pieces were subjected to serial incubations with EDTA to remove the epithelium, followed by digestion with collagenase to extract lamina propria lymphocytes. Peripheral blood and spleen cells were stained using a whole blood lysis technique. Blood, spleen, lymph node, and intestinal lymphocytes from all 45 animals were examined by four color flow cytometry with fluorescently conjugated monoclonal antibodies to CD4-APC (L200), CD8-PerCP (SK1), CD25-FITC (M-A251) or CD3-FITC (SP34-2) combined with CCR5-PE (3A9) or CD69-PE (FN50, BD Biosciences) in separate tubes. Samples were acquired on a FACS Calibur flow cytometer (Becton Dickinson) and analyzed with Flowjo software (Tree star, Inc.). To further characterize CD69+ CD4+ or CD8+ cells in blood and tissues, nine-color flow cytometry using appropriately diluted, directly conjugated monoclonal antibodies to CD45RA-FITC (5H9), CCR5-PE, CD95-PE-Cy5 (DX2), CD25-PE-Cy7, CD28-APC (28.2), CD69-APC-Cy7, CD3-Pacific Blue (BD Biosciences) CD8-PE-TR (3B5, Caltag Laboratories), and CD4-Qdot655 (L200, NIH) was performed on the same tissues from 5 normal macaques. These samples were resuspended with BD Stabilizing Fixative (BD Biosciences) and acquired on an LSRII flow cytometer (Becton Dickinson). Data were analyzed with Flowjo software (Tree star, Inc.).

### Immunohistochemistry and confocal microscopy

Three color immunofluorescent staining for CD69, CD4 or CD8 and CD3 (T cells) was performed on formalin-fixed, paraffin-embedded tissues from selected animals (uninfected and through day 21 of infection) to visualize and phenotype the distribution of CD69+ T cell subsets in tissues by confocal microscopy as previously described [33]. In brief, formalin-fixed, paraffin embedded sections were de-paraffinized and antigens “unmasked” using high temperature antigen retrieval consisting of heating slides in a steam bath chamber (Black and Decker Flavor Center Steamer Plus) with 0.01 M citrate buffer, pH 6.0 for 20 min, cooled, and washed twice in PBS.

Three-color fluorescent immunostaining was also compared on snap-frozen tissues. Briefly, intestinal tissues were rapidly frozen in optimum cutting temperature compound (Tissue-Tek® O.C.T. Compound, Sakura Finetek) submersed in dry-ice cooled 2-methylbutane, and 5 µm sections were fixed with cold acetone, then incubated with CD4 (1F6) or CD8 (1A5, Novocastra Laboratories Ltd.) or P28 (3F7, Trinity Biotech). Slides were then washed with PBS and incubated with Alexa Fluor 568 (red) labeled secondary antibody (goat anti-mouse IgG1, Invitrogen, Carlsbad, CA), for 30 min, washed and incubated with appropriately diluted primary antibodies, CD69 (CH/4, Invitrogen, Carlsbad, CA) and CD3 (rabbit polyclonal, Dako Inc.), washed, and incubated with Alexa Fluor 488 (green) labeled goat anti-mouse IgG2a (Invitrogen, Carlsbad, CA) and Alexa Fluor 633 labeled anti-rabbit IgG (H+L), (Invitrogen, Carlsbad, CA) secondary antibody to detect CD4 or CD8 (red) and CD3 (blue), respectively. Finally, slides were mounted with fluorescent mounting medium (Dako, Inc.) and visualized using a confocal microscope. Isotype mouse serum controls were performed on both frozen and paraffin-embedded slides and no false positive signals were detected for any of these markers. To determine the proportion of activated cells infected with SIV, lymph nodes and intestinal tissues from 3 macaques in primary infection (day 10) were sectioned and triple labeled as above for P28, CD3 and CD69, and the total number of SIV infected T cells (P28+CD3+) and SIV infected activated T cells (P28+CD69+CD3+) were counted and the proportion of infected activated T cells was calculated.

Confocal microscopy was performed using a Leica TCS SP2 confocal microscope equipped with three lasers (Leica Microsystems, Exton, PA). Individual optical slices represent 0.2 mm, and 32–62 optical slices were collected at 512×512 pixel resolution. NIH Image (version 1.62) and Adobe Photoshop (version 7.0) was used to assign colors to the channels collected.

### Statistics

Graphical presentation and statistical analysis of the data were performed using GraphPad Prism 4.0 (GraphPad Software Inc., SanDiego, CA). A Mann Whitney U test was used for comparison of CD69 expression between CD4+ and CD8+ T cells in tissues of normal macaques. Comparisons of CD69 expression in tissues during SIV infection were analyzed by a one-way ANOVA and Dunnett's Multiple Comparison Test to compare data from infected and control macaques (P<0.01). Correlations between samples were calculated and expressed using the Spearman coefficient of correlation.
